# Plasmatic carbonic anhydrase IX as a diagnostic marker for clear cell renal cell carcinoma

**DOI:** 10.1080/14756366.2017.1411350

**Published:** 2017-12-18

**Authors:** Laura Lucarini, Lucia Magnelli, Nicola Schiavone, Alfonso Crisci, Alessio Innocenti, Luca Puccetti, Fabio Cianchi, Sara Peri, Claudiu T. Supuran, Laura Papucci, Emanuela Masini

**Affiliations:** a Department of Neuroscience, Psychiatry and Drug Area and Child Health (NEUROFARBA), Section of Pharmacology and Toxicology and Section of Pharmaceutical Sciences, University of Florence, Florence, Italy;; b Department of Experimental and Clinical Biomedical Sciences “Mario Serio”, University of Florence, Florence, Italy;; c Experimental and Clinical Medicine, University of Florence, Florence, Italy;; d Unit of Urology, San Lazzaro Hospital, Cuneo, Italy;; e Department of Surgery and Translational Medicine, University of Florence, Florence, Italy

**Keywords:** Carbonic anhydrase, isoform IX, tumours, clear cell renal cell carcinoma

## Abstract

Carbonic anhydrase (CA, EC 4.2.1.1) IX is regarded as a tumour hypoxia marker and CA inhibitors have been proposed as a new class of antitumor agents, with one such agent in Phase II clinical trials. The expression of some CAs, in particular the isoforms CA IX and CA XII, has been correlated with tumour aggressiveness and progression in several cancers. The aim of this study was to evaluate the possibility that CA IX could represent a marker related to clear cell Renal Cell Carcinoma (ccRCC). Bcl-2 and Bax, and the activity of caspase-3, evaluated in tissue biopsies from patients, were congruent with resistance to apoptosis in ccRCCs with respect to healthy controls, respectively. In the same samples, the CA IX and pro-angiogenic factor VEGF expressions revealed that both these hypoxia responsive proteins were strongly increased in ccRCC with respect to controls. CA IX plasma concentration and CA activity were assessed in healthy volunteers and patients with benign kidney tumours and ccRCCs. CA IX expression levels were found strongly increased only in plasma from ccRCC subjects, whereas, CA activity was found similarly increased both in plasma from ccRCC and benign tumour patients, compared to healthy volunteers. These results show that the plasmatic level of CA IX, but not the CA total activity, can be considered a diagnostic marker of ccRCCs. Furthermore, as many reports exist relating CA IX inhibition to a better outcome to anticancer therapy in ccRCC, plasma levels of CA IX could be also predictive for response to therapy.

## Introduction

Renal cell carcinoma (RCC) accounts for about 3% of all solid malignancies^1^ shows the highest aggressiveness among all urologic neoplasms^2^. It is the seventh most common cancer in men and the ninth most common in women^3^. Incidence worldwide is about 209,000 new cases per year and 102,000 deaths per year. The incidence of all stages of this cancer has increased over several years, contributing to a steadily increasing mortality rate per unit population^3^. One-third of patients with RCC have evidence of metastatic disease at the time of diagnosis and about half of those treated by radical surgery eventually relapse. Metastatic RCC is usually resistant to conventional chemotherapy whereas only immunotherapy with interferon alpha (IF-α) or interleukin-2 (IL-2) has been demonstrated to provide significant results in terms of disease-free survival^4–6^.

Over 80% of sporadic RCCs are clear cell carcinomas (ccRCC) which have been extensively investigated as regards their molecular signatures. A high percentage of ccRCCs has been shown to be associated with the inactivation of the von Hippel–Lindau (*VHL*) tumour suppressor gene^7^. The *VHL* gene encodes a protein (pVHL) which is the substrate recognition component of a ubiquitin ligase complex targeting a protein transcription factor, hypoxia-inducible factor-1α (HIF-1α), for proteolysis^8^^,^^9^. Under hypoxic conditions, as well as in case of *VHL* inactivation, HIF-1α is stabilised and accumulates in the nucleus leading to subsequent over-expression of genes which are critical for tumour angiogenesis, glucose transport, epithelial proliferation, cell migration, and pH control^10^. Hypoxia in the tumour microenvironment is associated with poor prognosis and poor response to therapy, underlying the importance of studying the effect of potential anticancer drugs on the hypoxic pathway.

The carbonic anhydrase (CA, EC 4.2.1.1) family includes 16 catalytically active zinc metalloenzymes involved in the reversible hydration of carbon dioxide to bicarbonate and a proton^11^^,^^12^. These isoforms mainly differ in their catalytic activity, tissue distribution, and subcellular localisation. Indeed, there are cytosolic, mitochondrial, secreted, and membrane-bound isoenzymes^13^. Among the latter, CA IX and CA XII were found to be overexpressed in a wide variety of human tumours, being involved in cancer aggressiveness and progression^14^^,^^15^. In particular, CA IX is a downstream gene activated following either hypoxia (via HIF-1α) or *VHL* inactivation^16^. CA IX has extensively been investigated in ccRCCs and a high percentage of these tumours (up to 94%) express CA IX^17^^,^^18^.

Even if the main function of CA IX is to maintain intracellular pH homeostasis under hypoxic conditions that are common in solid tumours, it has been shown that CA IX also lowers E-cadherin-mediated cell adhesion^19^. Contrary to other tumours, CA IX over-expression in RCC is associated with good prognosis and improved response to immunotherapy^20^. This difference could be a reflection of molecular mechanisms responsible for CA IX expression: high CA IX expression in other tumours than ccRCC is hypoxia-related, whereas, is linked to *VHL* inactivation in ccRCC, where the latter is itself a marker of good prognosis^16^. The fact that CA IX is not detectable in normal kidney tissue suggests that detection of both CA IX protein and activity in plasma may be a useful diagnostic marker and a predictive tool to evaluate the effectiveness of therapy.

## Materials and methods

### Patients and tissue collection

Tissue samples were obtained from eight patients with ccRCC (seven males and one female) median age 68.5 (range 58–77 years) who underwent surgical resections at the Department of General Surgery, of Alba hospital and of University of Florence. Cancer tissue (from the edge of the tumour) and adjacent normal tissue (at least 10 cm from the tumour) were excised from each surgical specimen. All patients were thoroughly informed about the aims of the study and gave written consent for the investigation in accordance with the ethical guidelines of our University and of Alba General Hospital. None of the patients had taken nonsteroidal anti-inflammatory drugs for at least 3 months before surgery. All tumours were adenocarcinomas classified as: three patients stage I, five patients stage II according to the American Joint Committee on Cancer Staging System^21^; none had any colloid component. Samples were frozen at −80 °C for western blot analysis and at −20 °C for VEGF determination and caspase-3 activity evaluation until processing.

Plasma samples were obtained from 24 individuals: eight patients with benign tumours (six males and two females) median age 56.5 (range 44–77 years), eight healthy volunteers (five males and three females) median age 66.3 (range 54–68 years), eight with ccRCC (same patients as above) to evaluate CA IX plasma concentration and CA catalytic activity.

### Western blot analysis

Representative Western Blots were carried out in order to show CA IX, VEGF, pro-caspase 3, BAX, and BCL2 expression in normal and tumour tissue of patient with ccRCC. Human renal tissues were homogenised in RIPA buffer and incubated on ice for 1 h, and then centrifuged at 10,000*g* at 4 °C for 15 min. Following quantification by BCA assay kit (Thermo Fisher Scientific, Milan, Italy), 25 µg of total proteins from each supernatant were separated by SDS-PAGE under reducing conditions and then transferred to PVDF membrane. The membrane was blocked in 5% non-fat milk in 1% TBST, and then incubated overnight at 4 °C, respectively, with: 1:200 anti-CA IX monoclonal murine antibody M75^22^, recognising N-terminal extracellular PG domain of human CA IX. 1:1000 anti-VEGF monoclonal antibody; 1:1000 anti-procaspase-3 monoclonal antibody; 1:1000 anti-BAX monoclonal antibody; 1:1000 anti-BCL2 monoclonal antibody, were all from Santa Cruz Biotechnology (Los Angeles, CA).

Membrane-bound secondary antibodies (1:12,000) (Santa Cruz Biotechnology, Los Angeles, CA) were detected by ECL following the instructions of the manufacturer (Amersham, Freiburg, Germany). To ensure equal loading amounts, blots were stripped and reprobed using a 1:2000 monoclonal antibody raised against human tubulin (Los Angeles, Santa Cruz, CA).

### Semiquantitative reverse transcription-PCR (RT-PCR)

Total RNA was isolated according to the manufacturer’s protocol (NucleoSpin^®^ RNA II, Macherey-Nagel, Philadelphia, PA) and reverse transcribed (Omniscript, Qiagen, Milan, Italy) by using random primers. The RNA purity was validated by absorbance ratio. A typical PCR reaction (HotStarTaq, Qiagen, Milan, Italy) was prepared for amplification of CA IX mRNA, and calibration was performed by amplification of the same cDNA sample with primers for the housekeeping GAPDH mRNA. Primer sequences were as follows: CA IX 5-TAAGCAGCTCCACACCCTCT-3 (sense) and 5-TCTCATCTGCACAAGGAACG-3 (antisense), and the product size was 250 pb; for GAPDH, 5-GAGTCAACGGATTTGGTCGT-3 (sense) and 5-TTGATTTTGGAGGGATCTCG-3 (antisense) were used, giving a product size of 238 pb (Public Software Primer3; Whitehead Institute, Cambridge, MA). Amplification was performed as follow: 30 s of denaturation at 94 °C, 30 s of annealing at 56 °C, and 1 min of extension at 72 °C for 30 cycles. Amplification products were highlighted with ethidium bromide on 1.5% agarose gel. The intensities of the bands corresponding to the amplified products were quantified by densitometric analysis.

### Caspase-3 activity determination

The activity of caspase-3 was determined by use of a fluorescent substrate according to the method described^23^. The Ac-Asp-Glu-Val-Asp-AMC (Ac-DEVD-AMC; Bachem AG, Bubendorf, Switzerland) was used as a fluorescent substrate for caspase-3^24^. Tissues were homogenised with 10 mmol/L HEPES (pH7.4) containing 0.5% 3-[(3-cholamidopropyl)dimethylammonio]-1-propane-sulfonate, 42 mmol/L KCl, 5 mmol/L MgCl_2_, 1 mmol/L DTT, 1 mmol/L phenylmethylsulfonyl fluoride, 2 μg/mL leupeptin, and 1 μg/mL pepstatin A. The homogenate was then centrifuged at 10,000*g* for 10 min. Supernatants containing 250 μg of total protein were incubated with 40 μM of the caspase-3 substrate Ac-DEVD-AMC for 60 min at 37 °C. At the end of incubation, substrate cleavage was determined fluorometrically (Spectrofluo JY3 D, Jobin Yvon, Paris, France) with a *λ* excitation at 380 nm and a *λ* emission at 460 nm. The data were expressed as arbitrary units per milligram of protein. One unit enzyme activity was defined as the amount of the enzyme required to cleave 40 µmoles Ac-DEVD-AMC for 60 min at 37 °C. The determinations were done in quintuplicate.

### Determination of VEGF production

The concentration of VEGF in the tissues was measured using a commercially available ELISA kit according to the manufacturer’s instructions (Immunoassay Kit Human VEGF; Biosource International., Camarillo, CA). Samples were incubated in microtiter plates precoated with a monoclonal antibody specific for VEGF. After incubation at room temperature for 2 h and washing, a substrate solution was added. Colour development was stopped after 30 min at room temperature, and the colour intensity was read at 450 nm within 30 min. Protein concentration in the tissues was determined by the Pierce-BCA method (Thermo Fisher Scientific, Los Angeles, CA), using BSA as standard. Determinations were performed in triplicate and VEGF values were expressed as pg/μg protein.

### Determination of plasmatic concentration of CA IX

Plasmatic CA IX was measured using a quantitative sandwich enzyme immunoassay technique (Kit Quantikine**^®^** Human Carbonic Anhydrase IX/CA9 Immunoassay, R&D Systems, Inc., McKinley Place NE, Minneapolis, MN).

Plasma samples were washed three times with 0.9% NaCl and then haemolysed in cold water. The ghosts and any intact cell were removed by centrifugation at 15,000*g* for 10 min at 4 °C, and the pH of the haemolysate was adjusted to 7.4 with solid Tris-base. Samples absorbance was then measured at 280 nm.

### Determination of plasmatic activity of CA

An Applied Photophysics stopped-flow instrument has been used for assaying the CA catalysed CO_2_ hydration activity. Phenol red (at a concentration of 0.2 mM) has been used as indicator, working at the absorbance maximum of 557 nm, with 20 mM Hepes (pH 7.5), and 20 mM Na_2_SO_4_, following the initial rates of the CA-catalysed CO_2_ hydration reaction for a period of 10 s. The CO_2_ concentrations ranged from 1.7–17 mM for the determination of the kinetic parameters and inhibition constants. For each inhibitor at least six traces of the initial 5–10% of the reaction have been used for determining the initial velocity. The uncatalysed rates were determined in the same manner and subtracted from the total observed rates. Stock inhibitor solution (0.1 mM) was prepared in water and dilutions up to 0.01 nM were done. Inhibitor and enzyme solutions were preincubated together for 15 min at room temperature (prior to assay, in order to allow for the formation of the EI complex). The inhibition constants were obtained by non-linear least-squares methods using PRISM3, as reported earlier^25^ and represent the mean from at least three different determinations.

### Statistical analysis

Results are expressed as means ± SD. Multiple comparisons were performed by the Student test. Statistical significances were accepted at *p* < .05.

## Results

### CA IX expression in RCC

**Figure 1.**
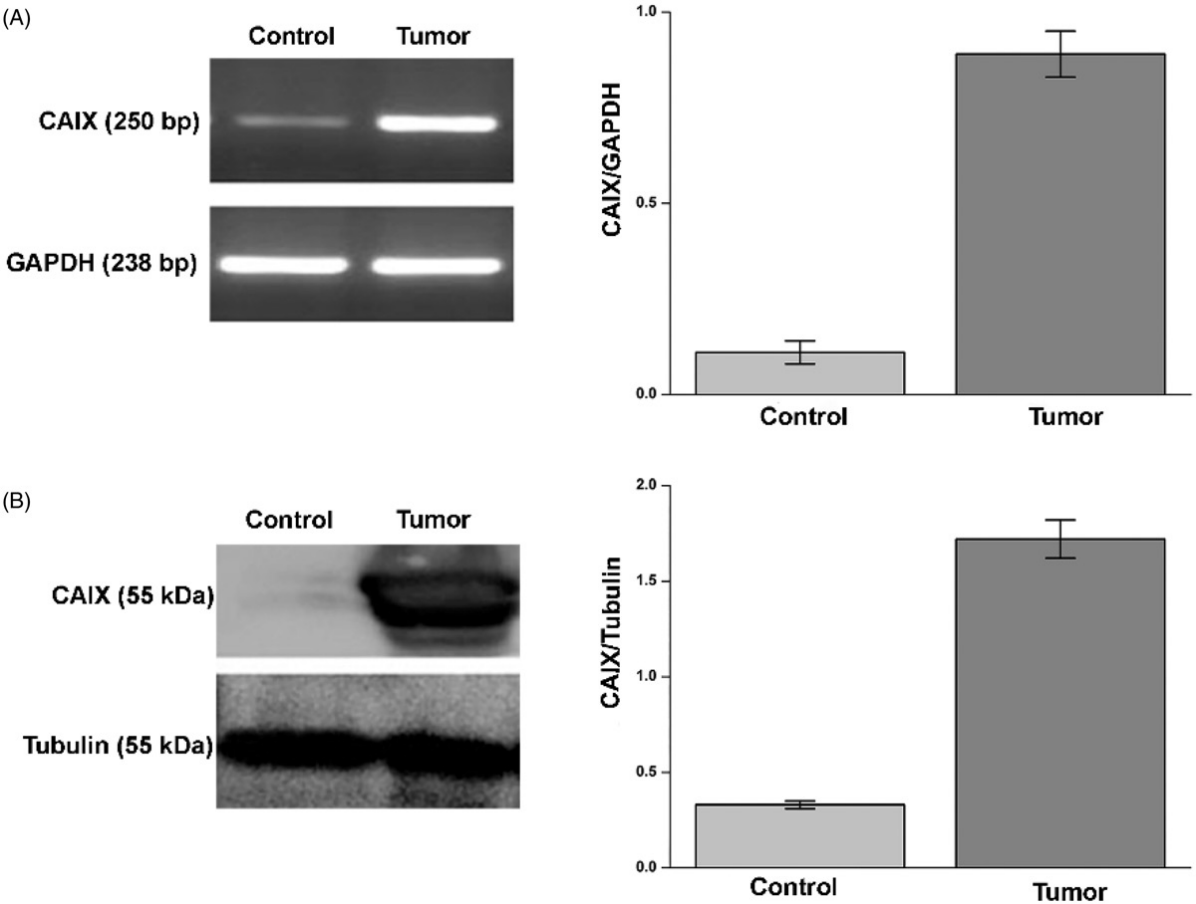
CA IX expression in ccRCC samples. mRNA levels (panel A, left side) evaluated by RT-PCR and protein levels (panel B, left side) evaluated by WB of CA IX in normal tissue (Control) and tumour sample (tumour) of a representative patient. GAPDH housekeeping gene amplification products and tubulin housekeeping protein were used to normalise values from RT-PCR and western blot, respectively; in right side of panel A (RT-PCR) and B (WB) are reported the densitometric values of samples from eight patients. Differences were significant for *p* ≤ .005 with t-test.

For each patient we determined the expression of CA IX both in tumour and corresponding normal tissue by semiquantitative RT-PCR and Western blot analysis. As expected, CA IX mRNA and protein were more expressed in tumour tissue compared to normal tissue (Figure 1, panels A and B).

### VEGF concentration

**Figure 2.**
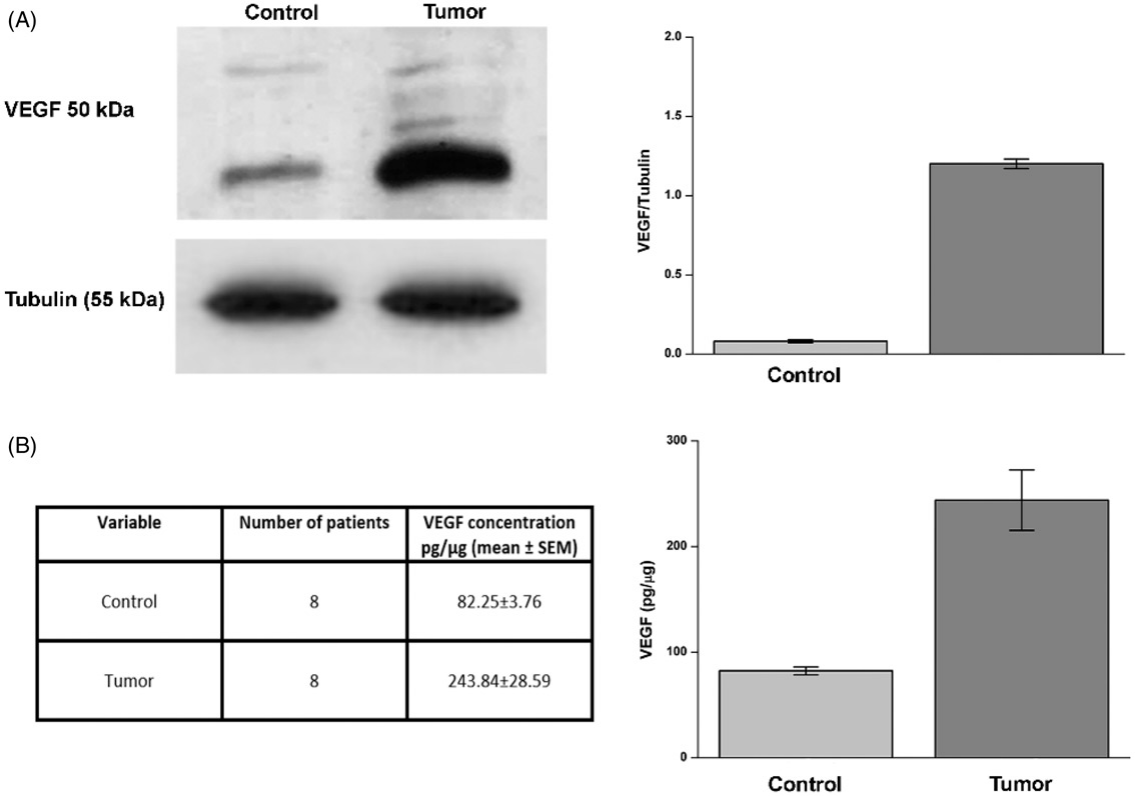
Evaluation of VEGF in ccRCC samples. Protein levels evaluated by WB (panel A) or ELISA (panel B) of VEGF in normal tissue (Control) and tumour sample (tumour). In panel A, right side, are reported the densitometric values of samples of WB from eight patients (differences were significant for *p* ≤ .005 with t-test). In panel A, left side, WB of VEGF from a representative patient is reported. Tubulin housekeeping protein was used to normalise values in WB. VEGF concentration by ELISA from same patients (panel B, left and right side) is expressed as pg/µg. Differences were significant for *p* ≤ .005 with t-test.

VEGF concentration has been evaluated in normal and matching tumour tissue from eight patients with ccRCC. Similarly to CA IX, VEGF is a downstream gene activated following hypoxia and/or VHL inactivation^10^^,^^26^. In tumour tissue, VEGF concentration was very high as assessed by Western blot and ELISA assays, with an average value of 243.84 pg/µg of protein, as compared to normal tissue where the VEGF average concentration was 82.25 pg/µg of protein (Figure 2, panels A and B).

### Apoptosis markers in ccRCC

**Figure 3.**
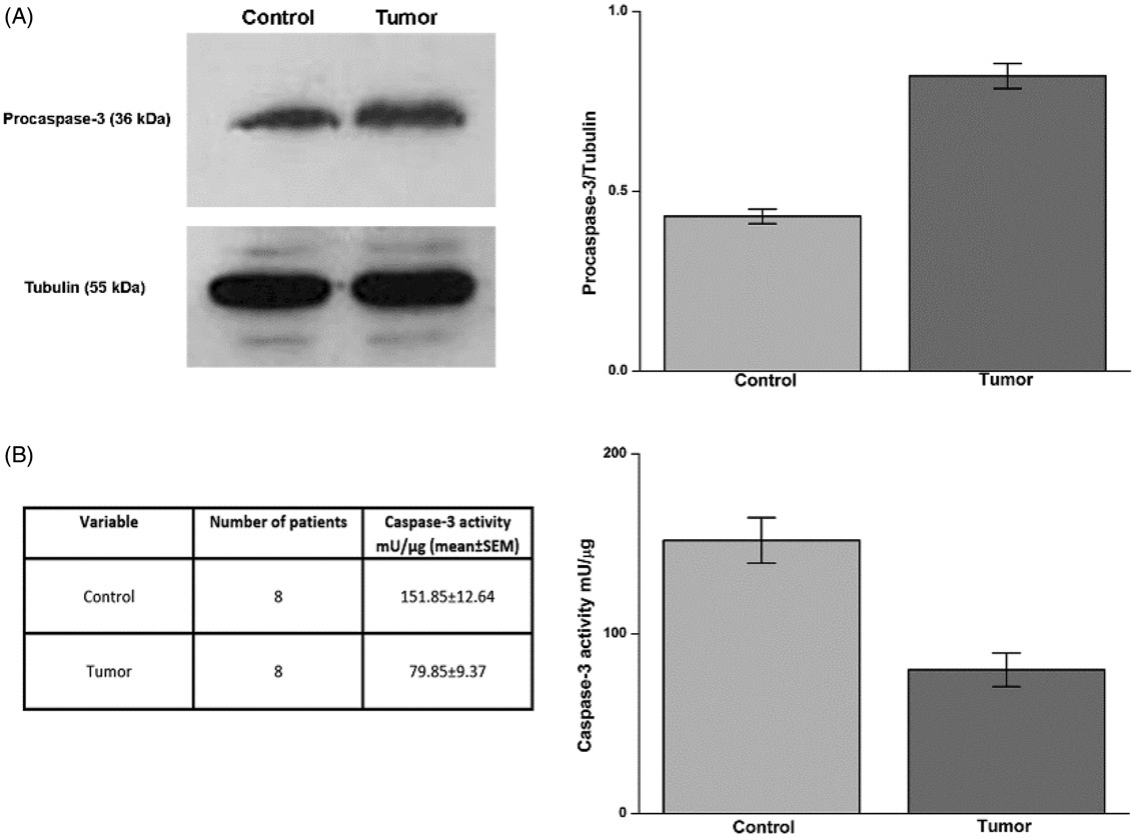
Procaspase-3 expression and caspase-3 activity in ccRCC samples. Protein levels evaluated by WB (panel A, left side) of Procaspase-3 in normal tissue (Control) and tumour sample (tumour) of a representative patient. Tubulin housekeeping protein was used to normalise values in WB. In panel A, right side, are reported the densitometric values of samples from eight patients. Caspase-3 activity in samples was evaluated as reported in material and methods and data are reported in panel B, left and right side. Differences were significant for *p* ≤ .005 with t-test.

We studied the expression of procaspase-3 by Western blot and caspase-3 activity by a fluorescent substrate in normal and tumour tissue from each patient. The expression of procaspase-3, the inactive precursor of the apoptosis effector caspase-3, was higher in tumour tissue in comparison to healthy tissue; accordingly, caspase-3 activity was significantly higher in the normal tissue (average activity 154.94 mU/µg) than in tumour tissue (average activity 79.85 mU/µg) (Figure 3, panels A and B).

**Figure 4.**
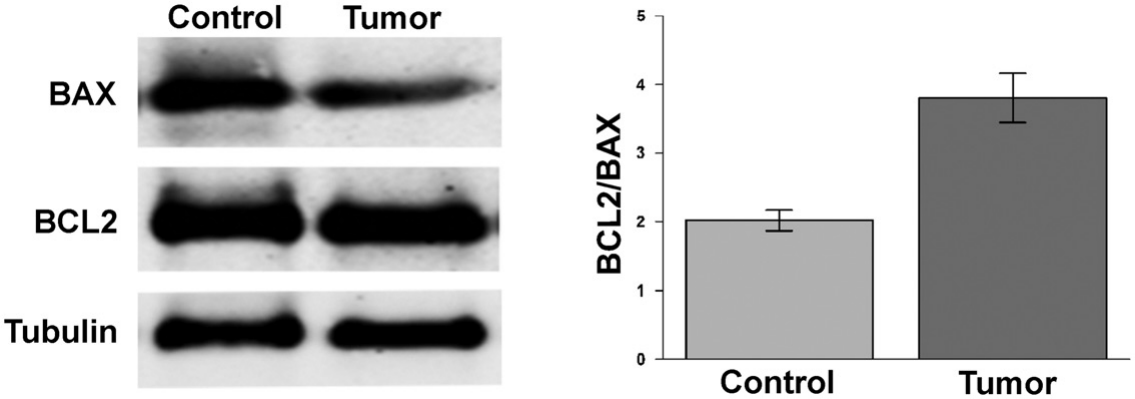
Ratio BAX/BCL2 in ccRCC samples. Protein levels of BCL2 and BAX evaluated by WB in normal tissue (Control) and tumour sample (tumour) of a representative patient. Tubulin was used to normalise values in WB. In left panel are reported the ratio between the values of BCL2 versus BAX. Differences were significant for *p* ≤ .005.

We analysed by Western blot the expression of the pro-apoptotic protein BAX and of the anti-apoptotic protein BCL2. The BCL2/BAX ratio in each renal biopsy analysed was higher in tumour tissue with respect to control tissue due to reduced BAX level (Figure 4).

### Plasma CA IX concentration and CA activity

**Figure 5.**
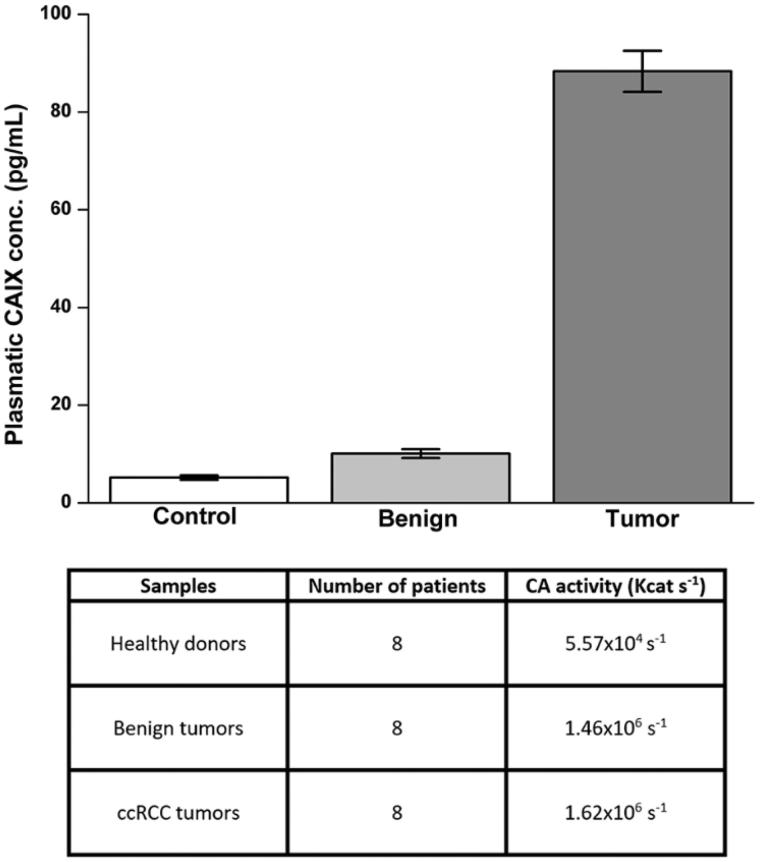
Evaluation of CA IX plasmatic concentration and CA enzymatic activity. Plasmatic concentration of CA IX evaluated by ELISA (upper panel). Plasmatic activity of CA (bottom panel) evaluated as reported in material and methods. Differences were significant for *p* ≤ .005 with t-test.

CA IX plasma concentration and CA activity were evaluated on plasma samples obtained by 24 patients (eight with ccRCC, eight with benign renal tumour, and eight healthy volunteers as described in Material and methods). The average concentration of CA IX in the plasma of patients with ccRCC was 93.48 pg/ml, a value significantly higher compared to healthy controls (6.23 pg/ml) and to patients with benign tumours (11.86 pg/ml) (Figure 5). There was a significant correlation between ccRCC and plasma CA IX concentration.

In the same samples of plasma the CA activity was evaluated. No particular differences were observed between the mean activity of CA in plasma samples of patients with ccRCC (kcat 1.62 × 10^6 ^s^−1^) and benign tumour (kcat 1.46 × 10^6^ s^−1^). On the contrary, the average value observed in plasma samples of healthy subjects was much lower (kcat 5.57 × 10^4^ s^−1^) (Figure 5). These results show that during tumuorigenesis, some mechanisms lead to an increase in plasma CA IX concentration, and an increase in CA catalytic activity even in benign tumours.

These data suggest that other CA isoenzymes in addition to CA IX could be up-regulated in non-malignant cancer, contributing to plasmatic total enzymatic activity.

## Discussion and conclusions

CA IX has been shown to be up-regulated in a number of human cancer cells as a consequence of either hypoxia-induced or constitutive HIF-1 activation, whereas, it is not expressed in their normal counterparts, with the exception of gastric mucosa^14^^,^^15^. Although these characteristics make CA IX an interesting target for novel approaches in the anticancer therapy, the exact role of CA IX in tumour growth and progression started only recently to be delineated. It has been shown that CA IX activity contributes to the acidification of hypoxic tumours through the decrease of the extracellular pH^27^^,^^28^. A low pH has been associated with tumorigenic transformation, chromosomal rearrangements, extracellular matrix breakdown, tumour cell migration and invasion^29^. Some new CA inhibitors with high affinity for CA IX isoforms have been shown to have potent antitumor activity in several cancer cell lines and one of them progressed to Phase II clinical trials recently^12^^,^^13^^,^^15^^,^^30^^,^^31^. However, the antiproliferative effect of CA inhibitors might be due to their effect not only on CA IX but on other CA isoenzymes, namely the other tumour-associated isoforms CA XII, CA II, and/or the mitochondrial isoform CA VA^32^.

In this study, we evaluated the possibility that CA IX could be a marker for the ccRCC diagnosis. In fact, we observed that CA IX is widely expressed in tumour tissue of patients with ccRCC as compared to healthy kidney tissue. CA IX probably plays an important role in regulating growth factors, such as VEGF and metalloproteinases which are involved in metastases. VEGF is highly expressed in the majority of RCCs^33^. Similarly to CA IX, VEGF is one of the HIF-1 target genes induced by either intratumoral hypoxia or *VHL* inactivation^34^^,^^10^. Accordingly, with *VHL* status, we found elevated VEGF levels in the ccRCC tissue samples as compared to normal tissue, and on this basis, we hypothesise that CA IX plays an important role in amplifying the VEGF signalling process by HIF-1α in ccRCC. VEGF may represent a marker of metastatic potential, and the signalling pathway of this growth factor represents a promising therapeutic approach for the treatment of metastatic RCC. As McIntyre et al.^35^ demonstrated, in colon cancer and glioblastoma, that inhibition of tumour hypoxic response by CA IX inhibition, potentiates the antiangiogenic therapy, we hypothesise that the same could be true also for ccRCC, highlighting CA IX as a therapeutic target alone or in combination with anti-VEGF therapy. Furthermore, in the same tumour samples, we observed both a reduced activity of caspase-3, and a decrease of BAX, shifting BCL2/BAX levels towards an antiapoptotic ratio. Hypoxia is a characteristic feature of locally advanced solid tumours resulting from an imbalance between oxygen supply and consumption. Tumour cell responses to hypoxic stress can be enhanced by proteomic and genomic changes, resulting in loss of apoptotic potential^36^. These new cell variants have advantages over less adapted cells in a hypoxic microenvironment and expand through clonal selection, often becoming the dominant cell type. Our findings clearly show that VEGF is abundantly expressed in tumour tissue, and VEGF over-expression together with CA activity could be correlated with the observed anti-apoptotic phenotype in ccRCC.

Evaluating the plasma concentrations in healthy subjects, patients with ccRCC and benign tumours, we observed that patients with ccRCC had a significant higher plasma concentration, whereas, no particular difference have been observed in total CA activity, between patients with ccRCC and benign tumours; while in healthy subjects, both CA IX concentration and CA activity were lower. We assume that the CA IX plasma concentrations, but not total CA activity, could be a marker of ccRCC. In spite of the relatively low number of samples, these results are homogeneous for each population and may open new prospectives for future, larger scale studies.
